# Report of a Case of Locomotor Attaxie Progressive, with Some Unusual Complications

**Published:** 1879-01-20

**Authors:** S. H. Anderson

**Affiliations:** Mo.


### *REPORT OF A CASE OF LOCOMOTOR ATTAXIE PROGRESSIVE; WITH SOME UNUSUAL COMPLICATIONS.*

BY S. H. ANDERSON, M. D., OF MO.

Called to see Andrew Thompson, May, 1878. Andy is a *dwarf*, weighing, perhaps, 90 pounds and aged 32 years. Andy is wrinkled, somewhat grey, and having the appearance of an old man of 60 or 70.

Having known Andy for a period of six years, his history, prior to present illness, is about as follows: When about seven years old Andy was attacked with high fever, delirium; great thirst, enormous diuresis; total loss of appetite; and, after an illness of several months, his present symptoms, which are those characterizing a well-marked case of the disease known to writers on medicine as "Locomotor Ataxie Progressive."

I am informed by Andy's mother that a daughter, when 7 years old, died in convulsions; and that another daughter when 13 years old committed *suicide* by *hanging*. A grown son, brother to Andy, has some *malformation* of the palate; and a married daughter is quite *deaf*. Father and mother appear to be healthy people. However, Mr.

Thompson informs me that *three* of his brothers have all died of some form of cancerous disease. A peculiarity of the family is, that when any member is laboring under fever of any kind, or from whatever cause, they are constantly and invariably *delirious*. The mother of Andy tells me that she is not aware of any cause giving rise to his disease as above described, unless it was this: Andy's father, without being what is called a drunkard, sometimes takes a *spree*, and, having been drinking, he had brought home a jug of whisky to which Andy had stolen during the night, and from which he had drunk so freely that he had become *beastly* drunk. Found, in fact, by his mother wholly insensible; and from which condition he could not be aroused for some twelve hours. At the time I was called to see Andy, rubeola was epidemic in the neighborhood; and, as expected, I found him laboring under a severe attack of the disease. Andy had been sick seven days when seen by the writer. Constant muttering delirium. No sign of the eruption. Pulse rapid and weak. Incontinence of urine and fæces.. Countenance of a deathly pallor and œdematous. Pupils dilated; extensive bronchitis; breathing rapid and difficult. On examination of chest, found the *heart* beating on right side of chest, low down. Called mother's attention to this malformation, when she stated that she had never known of its existence until his present illness. She had discovered the unnatural position of Andy's heart before I had called her attention to the matter.

Truly, I regarded the disease as beyond medical aid, and so stated to the parents. The merest tyro in medicine will recognize the gravity of the case, considered as an uncomplicated case of rubeola, without the superadded grave disease previously existing. Yet in order to gratify the parents, I consented to prescribe for Andy, as follows: ℞ Quinine sulph., grs. v; doveri, grs. iii; in decoction, rad. senega every three hours, to be alternated with v grs. iodide potass.; i. e. a dose of the first prescription, followed in one hour and a half by the potassie iodide. Blister 4x10 to be applied to nape of neck, extending well down between the shoulders. Directed Andy to have as much fresh sweet milk as he would take. Parents stated that alcoholic drinks crazed Andy, and therefore objected to their use. Saw Andy next morning at 10 a. m. Found my patient quiet and *sullen*, obstinately refusing to speak or answer any question whatever. However, I am inclined to think that Andy could not speak, as I believe that the nerves controlling the organs of speech were in a state of paralysis. Pupils dilated to some extent. Incontinence of urine and fæces have ceased. Respiration very slow and labored; not more, perhaps, than eight or ten per minute;—mayhap not so frequent. Was compelled to order attendant to sit constantly at his bedside, and to give him a shaking every now and then; otherwise, I believe he would have ceased to breathe. I recognized the fact that the functions of the *medulla oblongata* were seriously involved. Whether the result of changes years before, in the spinal cord, or whether the obtunding effects of the opium gave rise to this alarming symptom I could not decide. Perhaps both these causes were active factors. Ordered quinine and iodide potass. with senega decoction to be continued as before. Omitting the dover's powder. After omission of opiate from my prescription, Andy again became wildly delirious, with retention or suppression of urine. Parents being very ignorant people, objected to use of *catheter*, and prescribed, of their own accord, infusion of *maiden-hair*—bottles filled with warm water between the thighs, which had the desired effect.

Suffice it to say that Andy, after a protracted illness, recovered, contrary to all rational hopes, his usual health; and what is still more remarkable, since his recovery I have repeatedly made physical examinations of his chest, and find his heart beating in the *left* side, as is natural. Now, what caused this abnormal condition of Andy's heart during his illness ? Some will say, perhaps effusion in the *left* side of the chest. I can only say that, if so, I could not discover any such effusion. Hoping to hear some remarks on this, to me, very remarkable case, from some of my medical brethren, better informed than myself, I submit the imperfect report of the case.

